# Gene signatures of copper metabolism related genes may predict prognosis and immunity status in Ewing’s sarcoma

**DOI:** 10.3389/fonc.2024.1388868

**Published:** 2024-07-09

**Authors:** Yongqin Chen, Wencan Zhang, Xiao Xu, Biteng Xu, Yuxuan Yang, Haozhi Yu, Ke Li, Mingshan Liu, Lei Qi, Xiejia Jiao

**Affiliations:** ^1^ Department of Orthopedics, Qilu Hospital of Shandong University, Jinan, Shandong, China; ^2^ Sterile Supply Department, The First People Hospital of Jinan, Jinan, Shandong, China; ^3^ Department of Orthopedics, The Second Hospital of Shandong University, Jinan, Shandong, China

**Keywords:** Ewing’s sarcoma, copper metabolism related genes, prognostic-related copper metabolism related genes, functional enrichment, immune infiltration, risk model, clinical features

## Abstract

**Background:**

Cuproptosis is copper-induced cell death. Copper metabolism related genes (CMRGs) were demonstrated that used to assess the prognosis out of tumors. In the study, CMRGs were tested for their effect on TME cell infiltration in Ewing’s sarcoma (ES).

**Methods:**

The GEO and ICGC databases provided the mRNA expression profiles and clinical features for downloading. In the GSE17674 dataset, 22prognostic-related copper metabolism related genes (PR-CMRGs) was identified by using univariate regression analysis. Subsequently, in order to compare the survival rates of groups with high and low expression of these PR-CMRGs,Kaplan-Meier analysis was implemented. Additionally, correlations among them were examined. The study employed functional enrichment analysis to investigate probable underlying pathways, while GSVA was applied to evaluate enriched pathways in the ES (Expression Set). Through an unsupervised clustering algorithm, samples were classified into two clusters, revealing significant differences in survival rates and levels of immune infiltration.

**Results:**

Using Lasso and step regression methods, five genes (TFRC, SORD, SLC11A2, FKBP4, and AANAT) were selected as risk signatures. According to the Kaplan-Meier survival analysis, the high-risk group had considerably lower survival rates than the low-risk group(p=6.013e-09). The area under the curve (AUC) values for the receiver operating characteristic (ROC) curve were 0.876, 0.883, and 0.979 for 1, 3, and 5 years, respectively. The risk model was further validated in additional datasets, namely GSE63155, GSE63156, and the ICGC datasets. To aid in outcome prediction, a nomogram was developed that incorporated risk levels and clinical features. This nomogram’s performance was effectively validated through calibration curves.Additionally, the study evaluated the variations in immune infiltration across different risk groups, as well as high-expression and low-expression groups. Importantly, several drugs were identified that displayed sensitivity, offering potential therapeutic options for ES.

**Conclusion:**

The findings above strongly indicate that CMRGs play crucial roles in predicting prognosis and immune status in ES.

## Graphical Abstract

Flow chart. PR-CMRGs: prognostic-related copper metabolism related genes. DEGs, differentially expressed genes; GSVA, Gene Set Variation Analysis; PPI, protein-protein interaction.

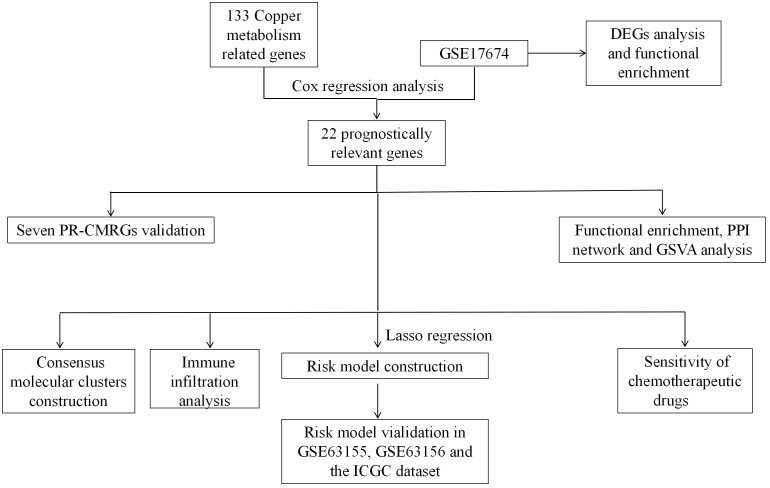

## 1 Introduction

Ewing’s sarcoma (ES) is a very aggressive cancer that mostly impacts the skeletal system and soft tissues in individuals who are in their childhood or teenage years (1). The documented prevalence of ES is 2.9 occurrences per million individuals annually (2). ES was distinguished by the merging of the EWSR1 gene with the FLI1 gene in previous researches (3, 4). The FLI1 gene belongs to the ETS gene group. A novel inhibitor of ETS proteins called TK216 has shown clinical benefits for almost half of the patients (5). The dedication of researchers and clinicians has advanced ES therapy. Traditionally, the main approach for treating ES has been a combination of surgery and radiation. However, despite comprehensive therapy, approximately 30–40% of patients continue to encounter recurrence or metastases. Less than 10% of ES patients with metastases still survive after five years. Currently, treating ES patients remains immensely challenging, necessitating the urgent identification of dependable intervention biomarkers to advance therapy.

Copper plays a crucial role in mitochondrial respiration, antioxidant defense, and programmed cell death (6). Maintaining an appropriate copper concentration is crucial for the survival of living organisms. Oxidative stress and cytotoxicity result from excessive copper levels, while copper deficiency can also be detrimental (7, 8). Cuproptosis is a novel kind of cellular demise induced by copper. In the tricarboxylic acid (TCA) cycle, copper ions bind to lipoylated components and engage in certain cellular interactions. This leads to the clustering of these copper-bound lipoylated proteins in the mitochondria, which in turn reduces the levels of iron-sulfur (Fe-S) clusters. This process causes proteotoxic stress and eventually leads to the death of the cell (6). Cu fills an essential function in the advancement and growth of cancer by stimulating the multiplication of cells, the formation of new blood vessels, and the spread of cancer cells to other parts of the body. Recent research indicates that the Cu-complex might be a promising target for cancer treatment. Cu has been found to induce apoptosis and/or the formation of free radicals, leading to the death of cancer cells (9, 10). Notable advancements in copper metabolism have been made in recent years. Several studies have revealed significant regulation of metabolism by proteins involved in copper handling and utilization in proliferating cells (11, 12). Cuproplasia, a novel kind of copper-dependent cell growth and proliferation, has been discovered (13). Both neoplasia and hyperplasia are included under this phrase. Growing evidence underscores the deep involvement of copper metabolism in cancer proliferation, angiogenesis, and metastasis (14, 15). Copper dysregulation is observed in cancer tissue than normal tissue (16–18). Various cancers have exhibited elevated amounts of copper (19). Additionally, copper imbalance is closely associated with weakened immune responses in cancer. Hence, a potential prognostic model based on CMRGs could effectively predict the prognosis and immune status of ES.

There is no study suggested that the mechanism of TFRC, SORD, SLC11A2, FKBP4, and AANAT in cuproptosis. In this study, we identified five signatures (TFRC, SORD, SLC11A2, FKBP4, and AANAT) based on CMRGs, which robustly predict ES prognosis. TFRC, SLC11A2, FKBP4, and AANAT were demonstrated associated with increased risk, while SORD was a favorable prognostic factor. Furthermore, we elucidated the correlation between these signatures and immune status. Ultimately, we concluded that CMRGs play a pivotal role in ES prognosis and immunity, providing valuable guidance for future research.

## 2 Manuscript formatting

### 2.1 Material and methods

#### 2.1.1 Data collection and preprocessing

Using the Molecular Signatures Database (MSigDB: https://www.gsea-msigdb.org/gsea/msigdb), we were able to identify 133 copper metabolism-related genes (**Supplementary Table 1**).Using the “GEOquery” software, we extracted profile matrices and clinical/survival data for this investigation from the GSE17674, GSE63155, and GSE63156 datasets in the Gene Expression Omnibus (GEO) database. We standardized all probe information to corresponding gene symbols based on annotation files using a consistent approach: when one gene symbol matched more than one probe, we eliminated the probes that had multiple gene symbols and chose the probe with the greatest expression value. GSE17674, comprised of 44 ES and 18 muscle samples, used the GPL570 platform. The GSE63155 and GSE63157 datasets, each containing 39 and 46 ES samples, respectively, were generated by the GPL5175 platform. Additionally, we obtained clinical data and expression profiles for 49 ES samples from the database of International Cancer Genome Consortium (ICGC). We designated GSE17674 as the training dataset, and GSE63155, GSE63156, and ICGC datasets as external test datasets. The flow chart of the study as follows (**Graphical Abstract**).

#### 2.1.2 Landscape of prognostic related CMRGs in ES

To assess the prognostic significance of CMRGs, we conducted univariate Cox regression analysis. We identified 22 CMRGs as prognostically relevant (PR-CMRGs). Using the “ggplot2” package, we visualized the results through a forest plot. Using the point_cut function to determine the cutoff value for PR-CMRGs, we classified the samples into categories based on their level of expression, distinguishing between high and low expression. We performed Kaplan-Meier analysis between these two expression groups using the “tinyarray” package. Utilizing the same package, we investigated the expression levels of PR-CMRGs in skeletal muscle and ES samples. To analyze correlations among these prognostic genes, we employed the “Pearson” method. The “corrplot” and “circlize” packages were used to present the correlation analysis results. We constructed a network of the 22 CMRGs using the “igraph” package and visualized the chromosomal positions of PR-CMRGs using the “RCircos” package.

#### 2.1.3 Functional enrichment, PPI network and GSVA

Using the “clusterProfiler” package, we performed functional enrichment analysis (GO/KEGG) to uncover potential mechanisms involving PR-CMRGs in ES. The results of the analysis were visualized using the ggplot package. To gain insight into potential interactions among PR-CMRGs, we identify protein interaction networks (with a minimum required interaction score > 0.150) by utilizing the STRING database (http://string-db.org/), and then depicted these results using the “igraph” package.

Gene Set Variation Analysis (GSVA) was conducted based on the “h.all.v7.5.1.symbols.gmt” gene set from the Molecular Signature Database. Using the “limma” package, we identified the differentially enriched scores of 50 pathways between ES and normal tissue, and illustrated these findings with bar plots.

#### 2.1.4 Construction of consensus molecular clusters

Based on the PR-CMRGs matrix to identify distinct molecular patterns that provide insight into the potential involvement of CMRGs in ES, unsupervised clustering was employed. For this task, we utilized the “ConsensusClusterPlus” package, known for its interpretability. We utilized PCA and t-SNE methods to confirm the differentiation of molecular clusters. To assess the survival differences among these clusters, we employed Kaplan-Meier curves. Additionally, we conducted an analysis of immune infiltration to gain insights into the status of the tumor microenvironment within these two clusters.

#### 2.1.5 Establishment and validation of risk model

To streamline the PR-CMRGs, we conducted Lasso regression analysis through the “glmnet” package. Utilizing the “survival” package, we then employed stepwise regression to identify the risk signature with the lowest Akaike’s information criterion (AIC). In addition, we used the “ggplot2” package to present the prognostic model via a forest plot.

The subsequent equation: the prognostic risk model was developed by conducting a study to calculate the risk score, which is determined by multiplying the Ei coefficient of a gene with its corresponding gene expression [risk score = Ei coefficient (gene i) × expression (gene i)]. Using the “survminer” package, the samples in the datasets were classified into high risk and low risk groups based on a predetermined cutoff value. Heatmaps, scatter plots, and PCA plots showed signature expression, survival status distribution, and risk score in different risk groups. Applying the Kaplan-Meier analysis with log-rank testing, survival curves were produced for these categories. Furthermore, using the “timeROC” package, the reliability of the risk model was confirmed by utilizing AUC values and time-dependent ROC curves.

Additionally, we evaluated the AUC of clinical features and risk model effectiveness in different clinical subgroups. Wilcoxon test was used to examine risk scores distribution in clusters and clinical subgroups. An alluvial diagram was employed to depict the link among risk groups, molecular clusters and clinical features.

#### 2.1.6 Establishment and validation of nomogram

We used Cox regression analysis to see if the risk score could act as a prognostic indication apart from clinical data(including gender, age, and stage status).Subsequently, using the “rsm” package, a nomogram was developed at risk level and clinical data, which facilitated prognosis prediction. Finally, evaluate the nomogram’s prediction accuracy at one, three, and five years, calibration curves were used.

#### 2.1.7 Analysis of immune infiltration

We utilized the ssGSEA method, implemented using the “GSVA” package to evaluate the differing amounts of 13 immunological pathways and 28 immune cells across groups classified as high and low risk. Additionally, we investigated immune cells variations in different signature expression groups.

We employed Spearman correlation analysis to explore how the risk score, different immune cells, and the signature were connected. These results were presented using lollipop diagrams created with the “ggplot” package. Lastly, we used the mantel approach in conjunction with the “ggcor” package to assess the link between the risk signature matrix and PR-CMRGs.

#### 2.1.8 Sensitivity of chemotherapeutic drugs

The “pRRophetic” package is designed for predicting clinical chemotherapeutic responses based on gene expression levels in various cancers, facilitating the evaluation of measured drug responses. The sensitivity of chemotherapeutic medications was tested across groups with high and low risk using this package. Using the expression profile of ES from the training dataset and expression data from Genomics of Drug Sensitivity in Cancer (GDSC, www.cancerrxgene.org/), we predicted the half-maximal inhibitory concentration (IC50) of drugs with a significance threshold of p < 0.001.

#### 2.1.9 Differentially expressed genes between risk groups, functional enrichment and GSVA

Using the “limma” package, we detected differentially expressed genes (DEGs) in different risk groups in the GSE17674 dataset (|LogFC| > 1, p < 0.05). For a deeper understanding of DEGs functions, based on |log2FC| > 1 and FDR value < 0.05, GO/KEGG analysis was conducted by using the “clusterProfiler” software. Visualization of the analysis results was done using the “ggplot” package. For KEGG pathways with |NES| > 1, NOM p value < 0.05, and FDR (p.adj) < 0.25, Gene Set Enrichment Analysis(GSEA) was conducted. Visualization of the results was accomplished using the “gseaplot2” and “ridgeplot” tools. We also conducted GSVA, comparing the enriched scores of pathways between risk groups using the Wilcoxon test. Heatmaps and boxplots were utilized to display the results.

### 2.2 Validation of Hub PR-CMRGs by RT-PCR

#### 2.2.1 Cell source

For this experiment, American Type Cell Culture (ATCC) provided RD-ES and A673 cells, and mesenchymal stem cells (MSCs) from Cyagen (Guangzhou, China).

#### 2.2.2 Real Time-PCR

Total RNA was extracted from cells using Trizol (Sigma, United States) and reverse transcribed into cDNA using a reverse transcription kit (Takara, Japan). The SYBR Premix Ex Taq (Takara, Japan) was utilized for real-time PCR, and the protocol provided by the manufacturer was adhered. Primer design was done using the “primerBank” website (pga.mgh.harvard.edu), and the primer sequences are provided in **Table 1**. The data analysis employed the 2-ΔΔCT method. At least three replications of each experiment were conducted.

**Table 1:** The sequences of the primers.

Gene Symbol	Forward primer	Reverse primer
DAXX	ACGTGCCCACTCTCTGTTTT	CAGAGGGCTCATTGGAGGTG
CCS	GGGAACTATTGACGGCCTGG	GTCAGCATCAGCACGGACAT
MTF2	GCATGTGGCGAAAAATACCG	GCAGTTGCTCCTTCCCATTC
F8	GGCCATCAGTGGACTCTCTTT	TAGCGAGTCAGTAACGGTGG
SLC11A2	GAGTGGTTACTGGGCTGCAT	CACAGGATGACTCGTGGGAC
IL1A	CTTCTGGGAAACTCACGGCA	AGCACACCCAGTAGTCTTGC
FKBP4	TGCTATCGTGGAGGTTGCAC	CTCCTTTCTCCATGCGCTGA

### 2.3 Statistical methods

R was utilized to execute bioinformatic analyses, and the Wilcoxon rank sum test was employed to analyze data between two groups. Spearman’s rank correlation was employed for the correlation study. Statistics were considered significant if P<0.05.R 4.1.3 (https://www.r-project.org) and GraphPad Prism 9.0 (GraphPad Software) were used for all statistical analyses.

## 3 Result

### 3.1 Landscape of prognostic related CMRGs in ES

**Figure 1:**
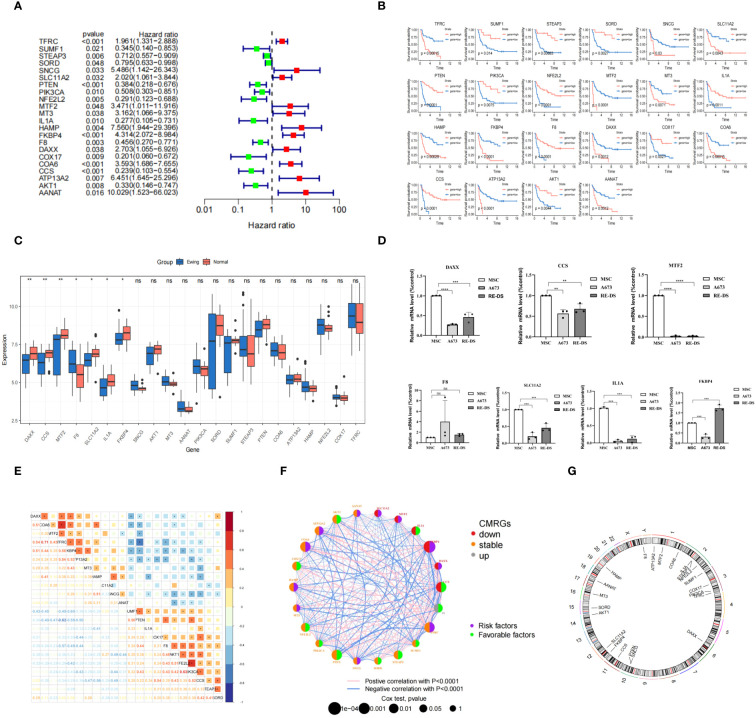
Construction of the CMRGS in ES and validation of hub PR-CMRGs. **(A)** The forest map of hazard ratios displayed the outcomes of univariate regression. **(B)** The result from the Kaplan-Meier analysis. **(C)** Boxplot of differentially expressed PR-CMRGs between Ewing and normal groups. **(D)** RT-PCR results of DAXX, CCS, MTF2, F8, SLC11A2, IL1A, and FKBP4. **(E, F)** PR-CMRGs correlation in ES. **(G)** Chromosomal positions of PR-CMRGs. * means p < 0.05, ** means p < 0.01, *** means p < 0.001, **** means p < 0.0001 and ns means no significance (p>0.05).

In our study, we investigated 133 CMRGs in GSE17674. Initially, the p-value and hazard ratio for each gene were calculated using univariate Cox regression. Subsequently, we identified 22 CMRGs with p-values < 0.05 as prognostically relevant genes (PR-CMRGs). A forest plot visualized the results, highlighting that among the 22 CMRGs, 11 genes including TFRC, SNCG, SLC11A2, MTF2, MT3, HAMP, FKBP4, DAXX, COA6, ATP13A2, and AANAT with HR > 1 were associated with worse survival (**Figure 1A**). The remaining 11 PR-CMRGs, which included SUMF1, STEAP3, SORD, PTEN, PIK3CA, NFE2L2, IL1A, F8, COX17, CCS, and AKT1 with HR < 1, were related to beneficial survival. Based on the levels of PR-CMRGs, the samples were divided into two groups: high expression and low expression. Kaplan-Meier analysis showed that there was a significant difference in survival rates between the two groups (**Figure 1B**).

Among the 22 PR-CMRGs, 7 (DAXX, CCS, MTF2, F8, SLC11A2, IL1A, and FKBP4) exhibited differential expression in the training dataset. Specifically, F8 was found to be enriched, while DAXX, CCS, MTF2, SLC11A2, IL1A, and FKBP4 were significantly down-regulated in ES samples compared to skeletal muscle tissue (**Figure 1C**).

RT-PCR results demonstrated that in A673 cells, the relative expression levels of seven hub PR-CMRGs were lower compared to MSCs, except for F8 which was higher. But the expression of F8 has not significant difference between them. In the RD-ES cell line, five of the hub genes were also significantly down-regulated in tumors, except for F8 and FKBP4 (**Figure 1D**).

Considering their biological function similarity, we explored the correlation among these PR-CMRGs. Notably, TFRC exhibited positive correlation with COA6 (r=0.71) while showing a negative correlation with PTEN (r=-0.62), underscoring TFRC’s central role in ES progression (**Figure 1E**).

The gene network of PR-CMRGs depicted their expression levels, correlations, and prognostic values in ES (**Figure 1F**). Chromosomal positions were visualized, revealing that F8 was located on the X chromosome, while the others were on autosomes. Chromosomes 1 (ATP13A2, MTF2, and COA6), 2 (IL1A, STEAP3, and NFE2L2), and 3 (COX17, PIK3CA, and TFRC) harbored the most genes, with no genes found on chromosomes 4, 5, 7, 8, 9, 13, 18, 20, 21, 22, and Y (**Figure 1G**).

### 3.2 Functional enrichment, PPI network and GSVA

**Figure 2:**
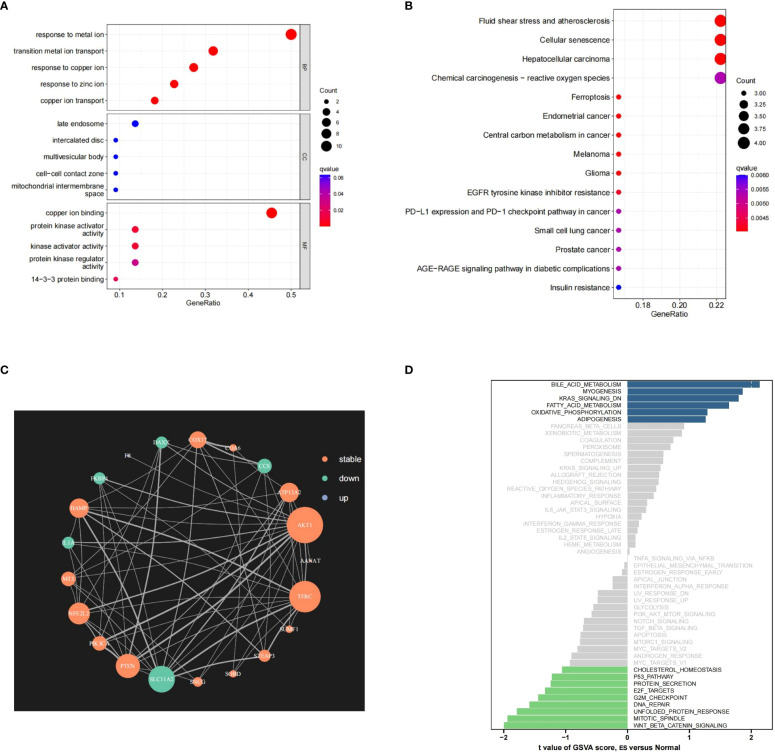
The outcomes of the enrichment analysis. **(A, B)** The outcomes of GO and KEGG study of PR-CMRGs. **(C)** The PPI network of PR-CMRGs. **(D)** Pathways alteration in ES by GSVA.

The PR-CMRGs were subjected to functional enrichment analysis, revealing involvement in processes like transition metal ion transport, response to copper ion and copper ion transport. In terms of cellular components, these genes were primarily located in the late endosome, intercalated disc, and multivesicular body. Molecular function analyses indicated participation in copper ion binding, protein kinase activator activity, and kinase activator activity (**Figure 2A**). KEGG analysis highlighted associations with various human cancers such as hepatocellular carcinoma, melanoma, and glioma. These enrichment results strongly emphasize the essential role of PR-CMRGs in carcinogenesis and cancer progression (**Figure 2B**). The PPI network of PR-CMRGs underscored the core positions of AKT1, TFRC, and SLC11A2 (**Figure 2C**). GSVA results demonstrated enriched scores for pathways like oxidative_phosphorylation, fatty_acid_metabolism, and kras_signaling_dn, with t-values>1,as well as pathways like wnt_beta_catenin_signaling,mitotic_spindle,andnfolded_protein_response with t-values < -1, when comparing ES and normal tissue (**Figure 2D**).

### 3.3 Consensus molecular clusters construction

**Figure 3:**
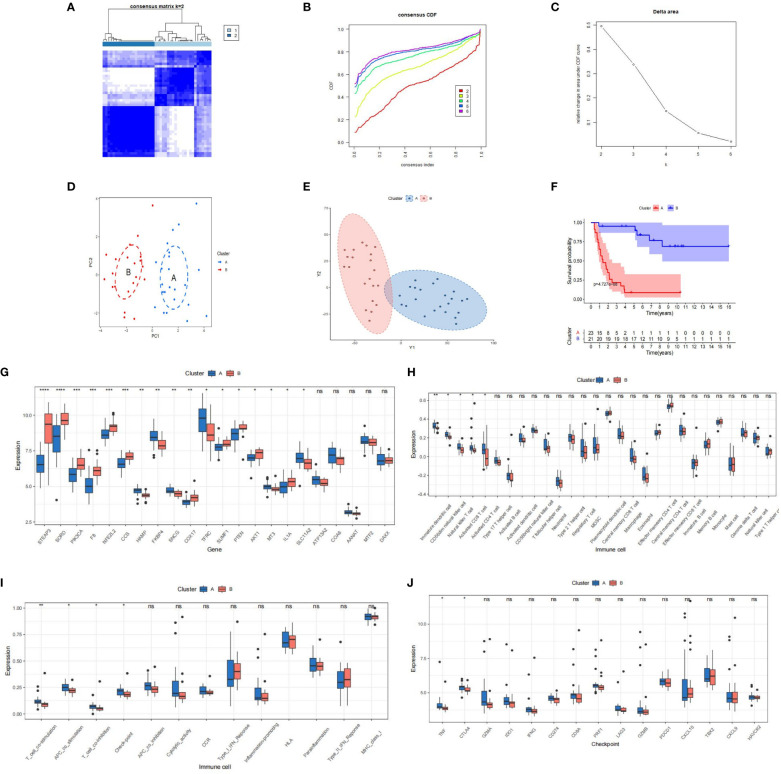
The outcomes of the consensus clusters, CRGs, and immune infiltration in clusters [**(A)**: blue vs. **(B)**: red] are as follows. **(A–C)** Two molecular clusters constructed using PR-CMRGs. The findings of **(D, E)** PCA and t-SNE show two distinct clusters. **(F)** Kaplan-Meier curves plotted for several molecular groupings. **(G–I)** A boxplot displaying the differential expression of PR-CMRGs and immune cells is shown. **(J)** A box diagram illustrating the distribution of checkpoint genes in clusters. * means p < 0.05, ** means p < 0.01, *** means p < 0.001, **** means p < 0.0001 and ns means no significance (p>0.05).

We conducted unsupervised clustering based on PR-CMRGs to discern distinct patterns. The optimal number of patterns was determined as K=2. Consequently, the 44 ES samples in GSE17674 were categorized into cluster A (n=23) and cluster B (n=21) (**Figures 3A-C**). The separation between these clusters was evident through PCA and t-SNE analyses (**Figures 3D, E**). In terms of survival advantage, cluster B outperformed cluster A, showing noticeably longer survival times.(p=4.727e−08) (**Figure 3F**). Dysregulated PR-CMRGs were observed in both clusters, as indicated by box plots (**Figure 3G**).

An analysis of immune infiltration unveiled that cluster A was characterized by higher levels of infiltration of Immature dendritic cells, Natural killer T cells, CD56dim natural killer cells, and Activated CD8 T cells (**Figures 3H, I**). Significant differences were observed between the two clusters in terms of immune function and checkpoints such as T-cell co-stimulation, APC co-stimulation, T-cell co-inhibition, and checkpoint genes (**Figure 3J**). Additionally, TNF and CTLA4 were enriched in cluster A. These findings collectively suggest that cluster A exhibited greater immunogenicity but worse prognosis.

### 3.4 Risk model construction and validation

**Figure 4:**
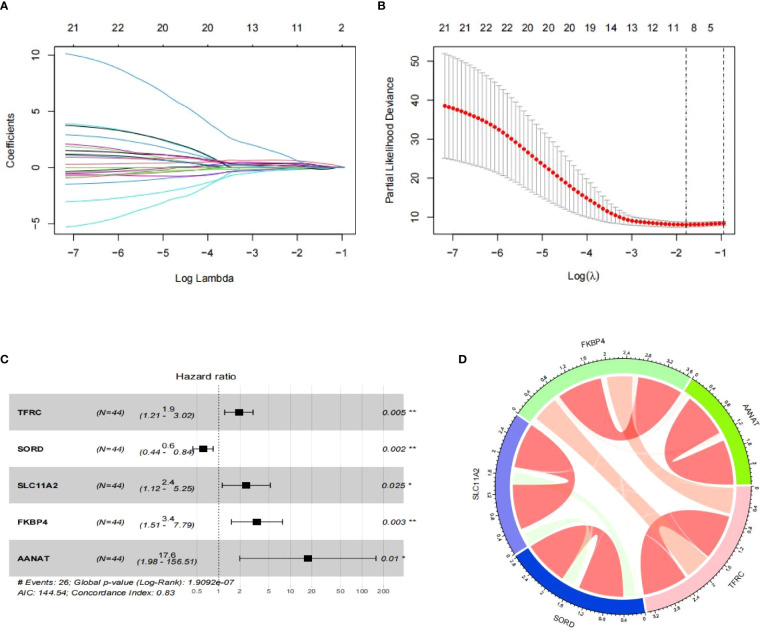
Development of a risk model. **(A, B)** The outcomes of Lasso regression. **(C)** Risk model depicted as a forest map. **(D)** Chord diagram of signature correlation. * means p < 0.05, ** means p < 0.01.

Utilizing Lasso regression, we reduced the number of PR-CMRGs to 10, and subsequently, 5 genes (TFRC, SORD, SLC11A2, FKBP4, and AANAT) were identified as the prognostic signature through stepwise regression, which yielded the lowest AIC (n=144.54) (**Figure 4A**). The forest plot visualization of the prognostic model indicated that TFRC, SLC11A2, FKBP4, and AANAT with HR > 1 were associated with worse survival, whereas SORD with HR < 1 correlated with favorable survival (**Figures 4B, C**). All signatures had p-values < 0.05, indicating the independence of each gene as a prognostic indicator. The correlation of the risk signature was depicted (**Figure 4D**).

The risk score prediction model was formulated as follows: RS = TFRC * 0.649 + SORD * -0.503 + SLC11A2 * 0.886 + FKBP4 * 1.232 + AANAT * 2.867. This algorithm was used to computed each sample’s risk score.

**Figure 5:**
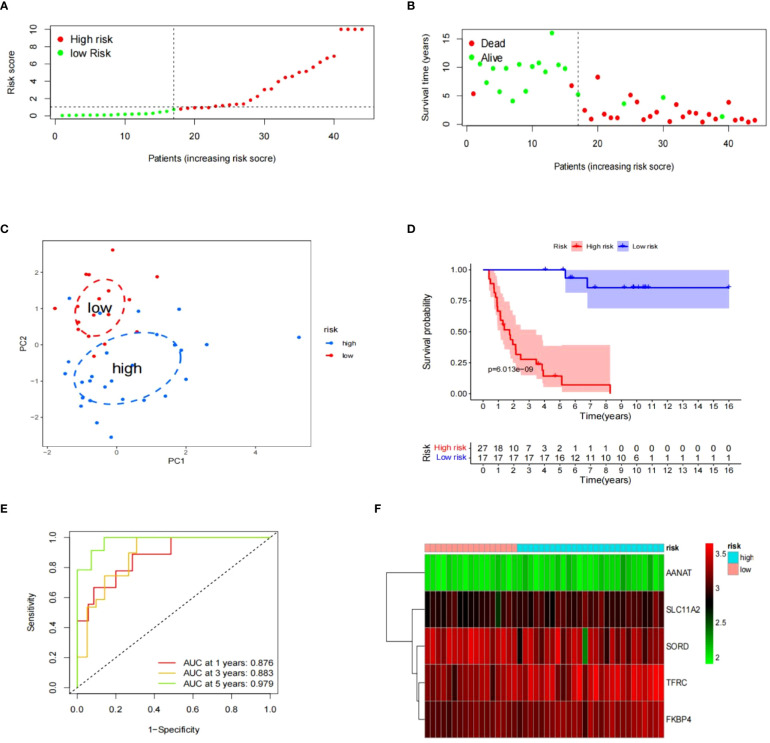
The prognostic significance of signatures in GSE17674. **(A–F)** The data includes the distribution of risk scores, survival status, PCA analysis results, K-M survival analysis results, time-ROC analysis results, and a heatmap of the signature.

The “surv_cutpoint” function was used to generate a cutoff value, which was then applied to split the ES samples in GSE17674 into two risk groups. Upon examining risk scores distribution and survival status, the high-risk group exhibited a more dismal prognosis than the low-risk group, which became evident. (**Figures 5A, B**). This distinction between risk groups was validated using the PCA method (**Figure 5C**). The survival curve for Kaplan-Meier with a p-value of 6.013e-09 indicated a significant correlation between risk groups and survival rates (**Figure 5D**). The time-dependent ROC curve with AUC values of 0.876, 0.883, and 0.979 at 1, 3, and 5 years respectively demonstrated the risk model’s strong performance in terms of specificity and sensitivity (**Figure 5E**). A heatmap was created to provide a clear visualization of the signature profile between the two groups (**Figure 5F**).

**Figure 6:**
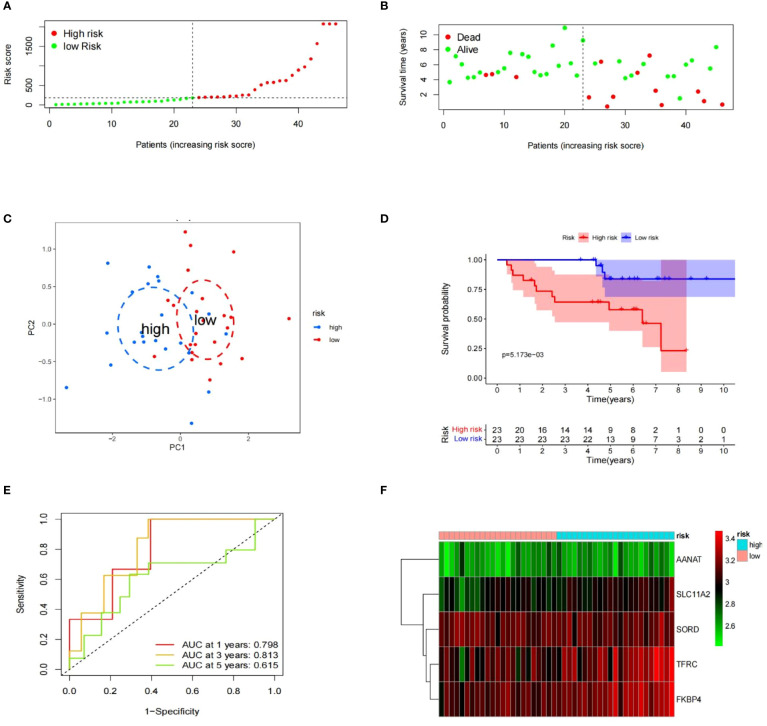
Prognostic value of signatures in GSE63155. **(A– F)** Distribution of the risk scores, survival status, PCA analysis, K-M survival analysis, time-ROC analysis, heatmap of the signature.

**Figure 7:**
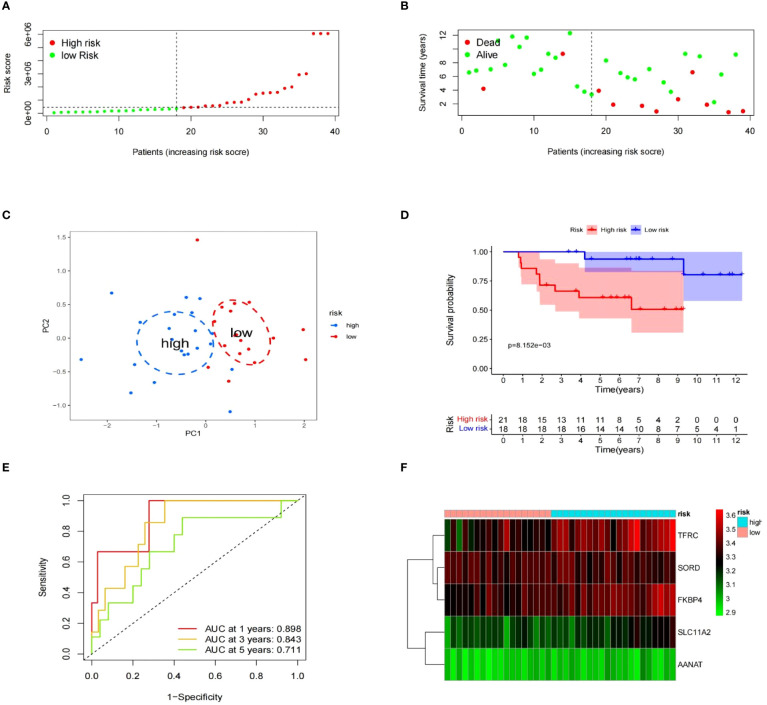
The prognostic significance of gene expression profiles in dataset GSE63156. **(A–F)** The information contains the distribution of risk scores, survival status, PCA analysis results, K-M survival analysis results, time-ROC analysis results, and a heatmap of the signature.

**Figure 8:**
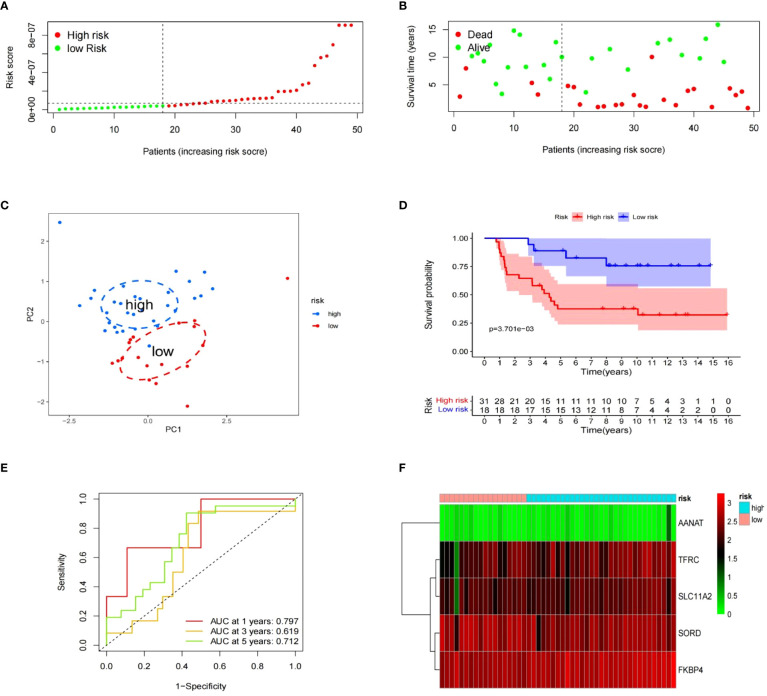
Value of signatures for prognosis ICGC dataset. **(A–F)** Risk score distribution, survival status, principal component analysis, K-M survival, time-ROC, and signature heatmap.

To evaluate the risk model’s accuracy, we utilized the signatures on three external datasets: GSE63155, GSE63156 and the ICGC dataset. Employing the RS formula, we calculated each sample’s risk score in the three test datasets. Samples were classified as different risk groups based on the cutoff value produced by the “surv_cutpoint” function (**Figures 6A, B**). Risk scores and survival status distribution was visualized, highlighting worse outcome in the high-risk group. The PCA plot confirmed the clear separation in the two groups (**Figure 6C**). Subsequently, time ROC curves, heatmaps and K-M survival curves were generated using methods similar to those in the training dataset (**Figures 6D-F**). A parallel conclusion was drawn through Kaplan-Meier survival analysis, revealing p-values of 5.173e-03, 8.152e-03, and 3.701e-03 in GSE63155, GSE63156, and the ICGC dataset, respectively. The timeROC results reflected commendable AUC values for 1, 3, and 5 years in GSE63155 (0.798, 0.813, 0.815), GSE63156 (0.898, 0.843, 0.711) (**Figures 7A-F**), and the ICGC dataset (0.797, 0.619, 0.712) (**Figures 8A-F**). These results substantiated the model’s ability to differentiate samples with favorable and worse prognoses, thereby demonstrating its potential for predictive prognosis.

### 3.5 Clinical features associated with risk model

**Figure 9:**
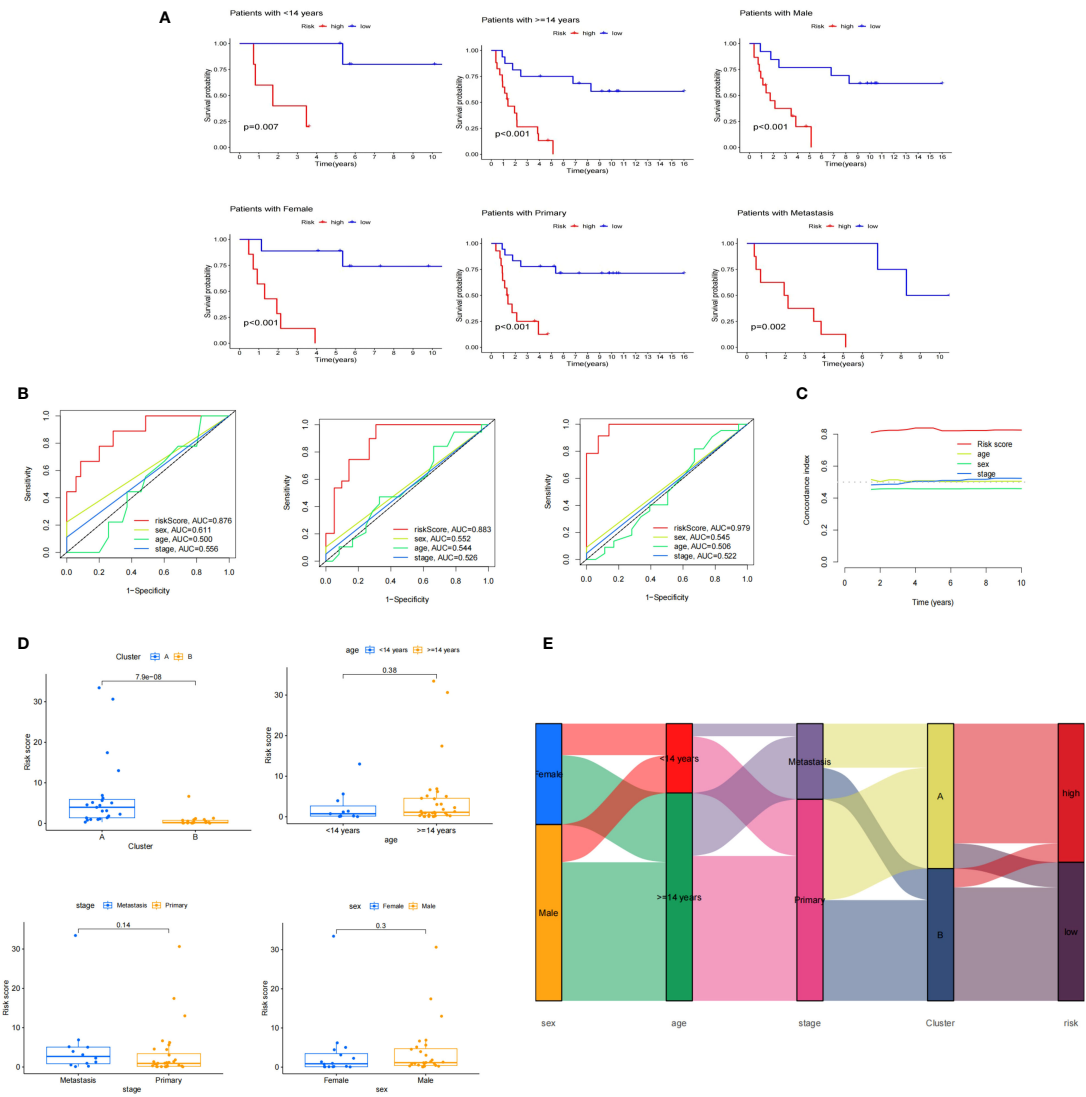
Clinical features associated with risk model. **(A)** Perform K-M survival analysis on age, sex, and stage subgroups; **(B)** Conduct ROC analysis to evaluate the risk score and clinical features (sex, age, stage) at 1, 3, and 5 years; **(C)** Calculate the C-index for the risk score and clinical features (sex, age, stage); **(D)** Examine the distribution of the risk score among clusters and clinical subgroups; **(E)** Create an alluvial diagram to visualize the relationship between molecular clusters, risk groups, and clinical features.

Using Kaplan-Meier survival method, we tested the risk model’s prediction effectiveness across different clinical subgroups. It demonstrated that the risk model was effective in predicting prognosis for certain subgroups, including individuals aged 14 years and older (p<0.001), individuals less than 14 years (p=0.007), females (p<0.001), males (p<0.001), individuals with primary stage cancer (p<0.001), and those with metastatic stage cancer (p=0.002) (**Figure 9A**). The fact that risk scores produced the greatest AUC values was confirmed by ROC curves that plotted risk scores against clinical variables (age, sex, and stage) for1,3, and 5 years (**Figures 9B, C**). Furthermore, a box plot was employed to display the distribution of risk scores across different molecular clusters and clinical subgroups (**Figure 9D**). An alluvial diagram showcased that samples associated with females, adults, metastasis, and cluster A exhibited elevated risk scores (**Figure 9E**).

### 3.6 Establishment and validation of nomogram

**Figure 10:**
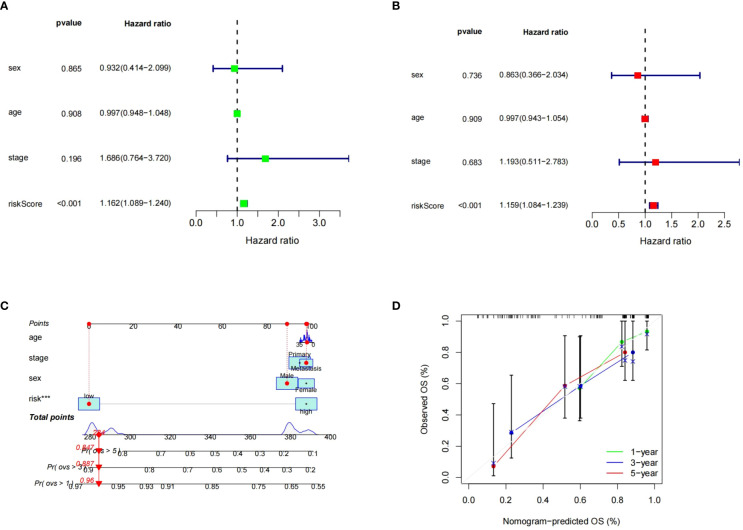
Independence of the risk model in GSE17674. **(A, B)** The results of univariate and multivariate COX regression analysis. **(C)** Nomogram for predicting 1, 3 and 5-year OS. **(D)** The calibration plots for predicting 1, 3, 5-years OS. *** means p < 0.001.

To assess the risk score prognostic independence, Cox regression analysis was performed. According to both the univariate and multivariate regression analysis, the risk score significantly affected ES prognosis, indicating that it was an independent prognostic factor (p<0.05).While no significant prognostic associations were observed with clinical features (**Figures 10A, B**). Subsequently, the risk level and clinical features were used to build a predictive nomogram. (**Figure 10C**). The 1-, 3-, and 5-year calibration curves showed that the ideal and predictive curves were well aligned (**Figure 10D**).

### 3.7 Immune infiltration analysis

**Figure 11:**
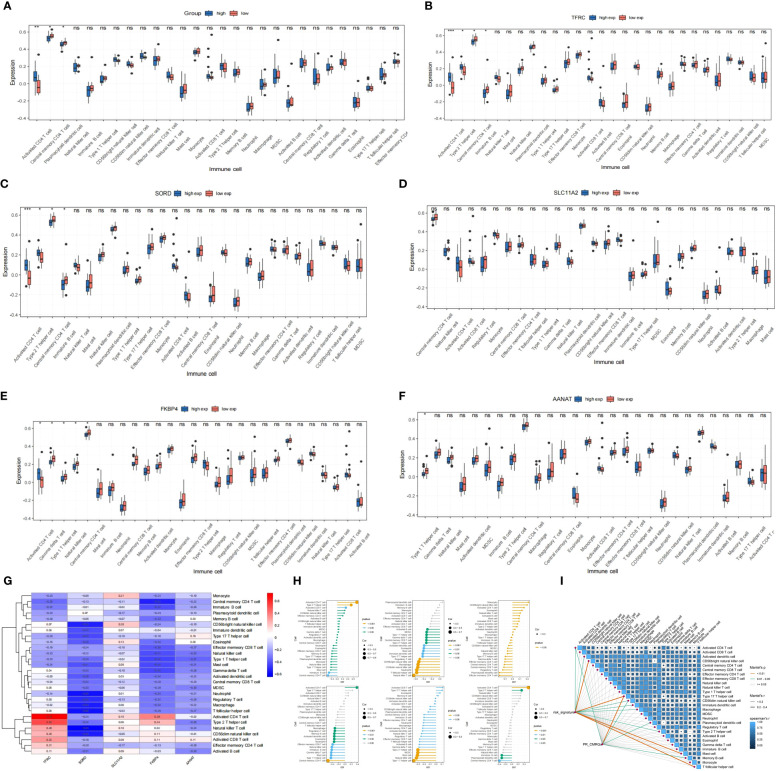
**(A)** Evaluation of immune cell infiltration between high and low risk groups (high: blue vs. low: red). **(B–F)** Boxplot of immune cell infiltration in differential TRFC,SORD,FKBP4,AANAT and SLC11A2 expression groups(high: blue vs. low: red). **(G–I)** Correlation of signatures and infiltrated immune cells, immune related pathways. * means p < 0.05, ** means p < 0.01, *** means p < 0.001 and ns means no significance (p>0.05).

Using ssGSEA analysis, we determined the abundance of different immune cells. The results showed that whereas activated CD4 T cells were more common in the high-risk group, central memory CD4 T cells and plasmacytoid dendritic cells were much more prevalent in the low-risk group (**Figure 11A**).

Further analysis indicated that Type 2 T helper cells and Activated CD4 T cells were significantly increasing in the high-expression group of TRFC and SORD, whereas Central memory CD4 T cells and Immature B cells were more abundant in the group with reduced expression of TRFC and SORD. In the case of FKBP4, the low-expression group exhibited enrichment of Gamma delta T cells, Type 1 T helper cells, Natural killer cells, and Central memory CD4 T cells. On the other hand, the group with high expression had elevated quantities of activated CD4 T cells. Low-expression of AANAT was associated with enriched Type 1 T helper cells. SLC11A2 groups did not exhibit differences in immune cells (**Figures 11B-F**) (**Table 2**).

**Table 2:** Differential immune cell infiltration in differential TRFC,SORD,FKBP4,AANAT and SLC11A2 expression groups.

	TFRC		SORD		SLC11A2		FKBP4		ANNAT	
	High expression	low expression	High expression	low expression	High expression	low expression	High expression	low expression	High expression	low expression
Activated CD4 T cell	up		up				up			
Central memory CD4 T cell		up		up				up		
Gamma delta T cell								up		
Immature B cell		up								
Natural killer cell				up				up		
Type I Thelper cell								up		up
Type 2 Thelper cell	up	up								

The heatmap and lollipop plots highlighted correlations between immune cells and signatures (**Figures 11G-I**). Notably, the association between TFRC and Activated CD4 T cells was positive, with the highest r=0.73, while SORD displayed a negative correlation with T follicular helper cells and Neutrophils with r=-0.58. These findings imply a potential collaboration between risk signatures and immune cells in influencing ES clinical prognosis.

The findings presented above lend support to the notion that samples in the low-risk group exhibit better prognoses, likely due to heightened immune cell infiltration. These results offer potential evidence for the feasibility of immunotherapy in ES. However, further validation through additional studies is necessary to solidify these findings.

### 3.8 Sensitivity of chemotherapeutic drugs

**Figure 12:**
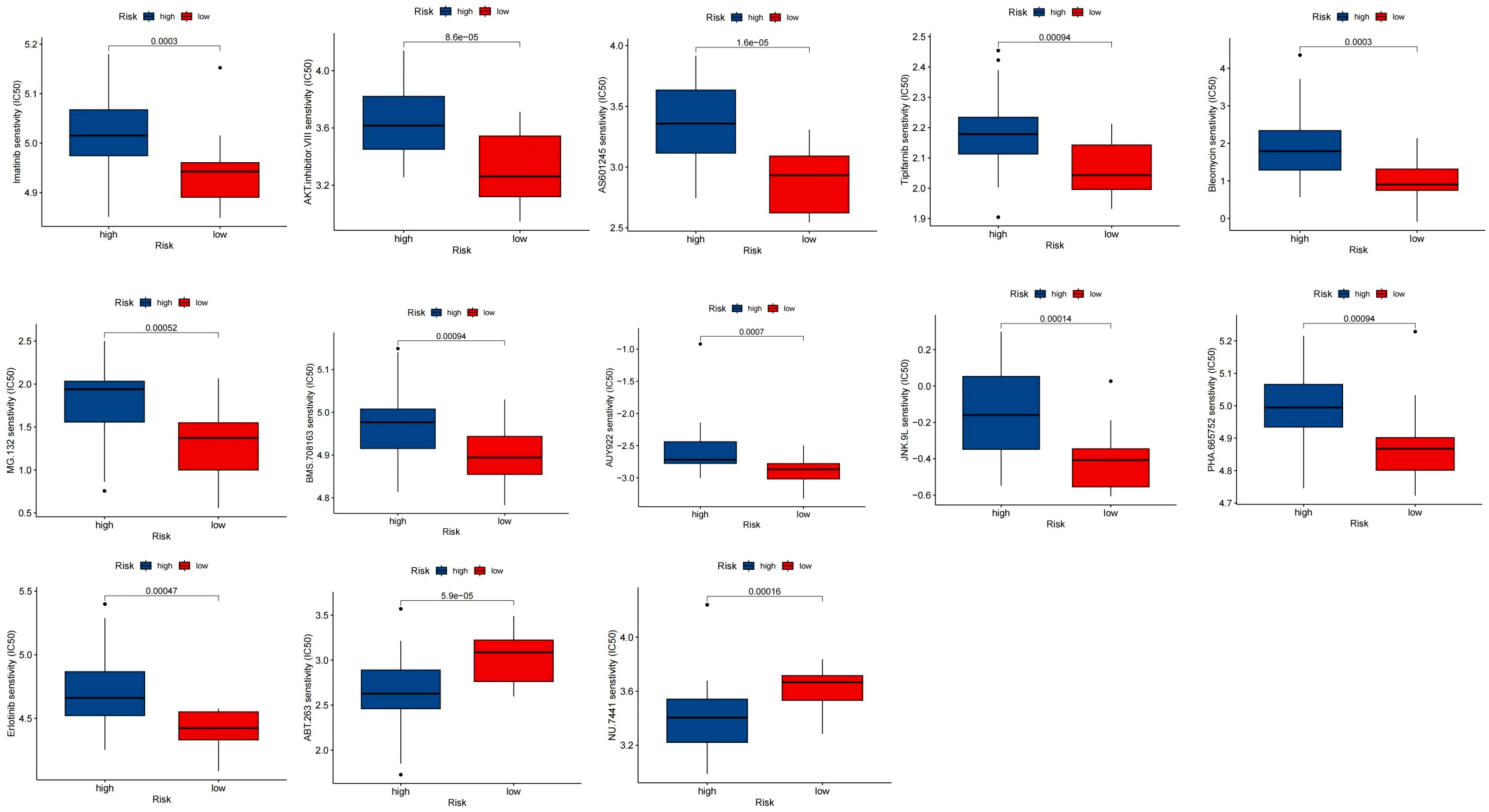
Chemotherapeutic response between risk groups. Boxplot of drugs benefited for samples in high and low risk groups.

Employing the “pRRophetic” package, clinical data was used to investigate the differential chemotherapeutic response in different risk groups. The findings showed that NU.7441 and ABT.263 were expected to be beneficial for the low-risk group, whereas exhibited greater benefit from AKT.inhibitor.VIII, AS601245, AUY922, Bleomycin, Tipifarnib, PHA.665752, MG.132, JNK.9L, BMS.708163, Erlotinib, and Imatinib in the high-risk group (**Figure 12**).

### 3.9 Differentially expressed genes between risk groups and functional enrichment

**Figure 13:**
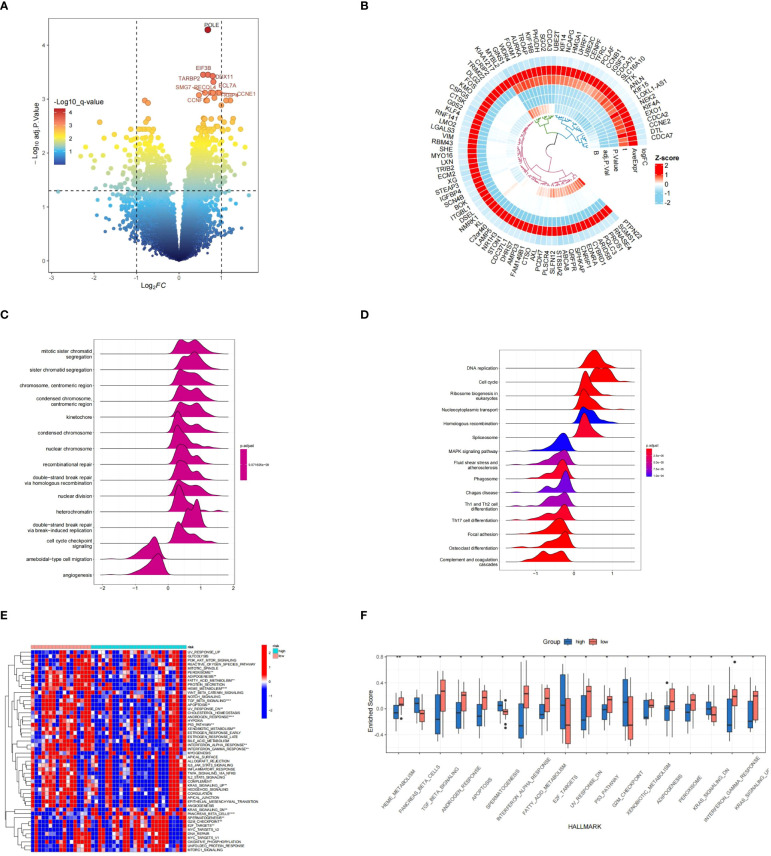
Analysis of differentially expressed genes (DEGs) and functional enrichment results. **(A, B)** Visualization of DEGs between various risk categories using a volcano plot and heatmap. **(C, D)** A ridgeplot displaying the GO and KEGG data by GSEA. **(E, F)** The results of GSVA analysis in groups classified as high and low risk. * means p < 0.05, ** means p < 0.01.

A total of ninety genes that exhibited differential expression were discovered in the GSE17674 dataset, visualized through volcano and circular heatmap representations (**Figures 13A, B**).GO terms like Mitotic sister chromatid segregation, sister chromatid segregation, and chromosome, centromeric region were shown to be prevalent, according to functional enrichment analysis. In terms of KEGG pathways, the analysis was primarily associated with DNA replication, Cell cycle, and Ribosome biogenesis in eukaryotes (**Figures 13C, D**).

The GSVA results indicated that the high-risk group exhibited pathways enrichment, including pancreas_beta_cells,spermatogenesis,e2f_targets,g2m_checkpoint,ras_signaling_dn, and others. Conversely, pathways like heme_metabolism,tgf_beta_signaling,androgen_response,apoptosis,interferon_alpha_response,fatty_acid_metabolism,uv_response_dn,p53_pathway,xenobiotic_metabolism,adipogenesis,peroxisome,interferon_gamma_response,and kras_signaling_up,pancreas_beta_cells,spermatogenesis, e2f_targets, g2m_checkpoint, ras_signaling_dn were more prevalent in the group at low risk. (**Figure 13E**). Byheme_metabolism,tgf_beta_signaling,androgen_response,apoptosis,interferon_alpha_response,fatty_acid_metabolism,uv_response_dn,p53_pathway,xenobiotic_metabolism,adipogenesis,peroxisome,interferon_gamma_response,kras_signaling_up,and spermattogenesis e2f_targets g2m_checkpoint kras_signaling_dn were showed in the high risk group (**Figure 13F**).

## 4 Discussion

The prognosis of ES remains poor despite comprehensive treatment strategies (20). Proto-oncogenes and tumor suppressor genes undergo a number of genetic and epigenetic modifications over the course of Ewing’s sarcoma development (21). As a vital micronutrient, copper is involved in many different physiological processes. According to earlier studies, altering copper metabolism may prevent the growth and invasion of cancer cells (22). Additionally, there have been the development of therapeutic strategies that specifically focus on copper or proteins involved in copper metabolism (23, 24).This work aims to examine the function of CMRGs in ES, Given the significance of copper in cancer.

The purpose of this study is to investigate the prognostic potential of CMRGs in ES. Using univariate Cox regression, we identified 22 CMRGs as prognostic indicators. The Kaplan-Meier analysis revealed notable disparities in survival rates between the groups with high and low expression of these PR-CMRGs. Notably, seven PR-CMRGs—DAXX, CCS, MTF2, F8, SLC11A2, IL1A, and FKBP4—displayed differential expression in ES samples. Moreover, the expressions of seven PR-CMRGs were valided in MSCs, RD-ES and A673.Functional enrichment analysis, encompassing GO and KEGG pathways, revealed key functions and pathways through which these PR-CMRGs impact ES prognosis. These included responses to metal ions, transition metal ion transport, copper ion binding, and pathways related to various human cancers. These findings emphasized the link between copper metabolism and the formation and advancement of cancer.

To comprehend the molecular subtypes of ES based on PR-CMRGs, unsupervised clustering identified two distinct molecular clusters with differing survival rates. Additionally, these clusters exhibited distinct immune cell infiltration patterns, highlighting the potential involvement of the immune system in ES prognosis.

Prognostic risk models provide valuable insights into predicting cancer results. Such models can offer information about survival likelihood, risk of recurrence, and potential treatment responses, assisting clinicians in making well-informed treatment decisions. Given the significant role of CMRGs in ES, a risk model was established based on these genes.

There is no study suggested that the mechanism of TFRC, SORD, SLC11A2, FKBP4, and AANAT in cuproptosis. But in this study, it is demonstrated that TFRC, SORD, SLC11A2, FKBP4, and AANAT associated with survival and immune infiltration. The potential mechanism need further research. In this study, we identified TFRC, SORD, SLC11A2, FKBP4, and AANAT as a risk signature for ES. The prognostic model constructed using these genes demonstrated that TFRC, SLC11A2, FKBP4, and AANAT, each with a HR greater than 1, were associated with increased risk, while SORD, with an HR less than 1, was a favorable prognostic factor. The efficacy of the risk signature was comprehensively assessed through the utilization of Kaplan-Meier survival and ROC curves in training dataset and three validation datasets. The correlation between the risk group and survival rates was consistently observed, and the signature’s specificity and sensitivity were robustly validated.

The TFRC gene in humans is responsible for encoding the transferrin receptor protein 1, controlling intracellular iron levels. Increased expression of TFRC promotes ferroptosis during CVB3 infection by recruiting Sp1 to the nucleus (25). TFR1, which mainly regulates cellular iron intake (26), binds to transferrin that is laden with iron, becomes surrounded by vesicles coated with clathrin, and then gets taken up by cells (27). TFR1 imports extracellular iron into cells, supporting the cellular iron store and being essential for ferroptosis (28). There is a close relationship between the iron and copper metabolic fares. The study demonstrated that the presence of Cu and Zn hindered the absorption of Fe, whereas the presence of Fe hindered the absorption of Cu (29). Thus, TFRC maybe change homeostasis of cellular iron to intervene copper metabolism. SORD is a dehydrogenase/reductase protein and plays a role in the metabolism of glucose (30). Studies have indicated SORD’s involvement as a cuproptosis-related gene in coronary artery disease (31). SLC11A2 is a key protein that aids the absorption of iron and its influence extends to breast and colon cancer progression (32, 33). Divalent metal ion transporter 1 (DMT1; often referred to as SLC11A2) has the ability to transport several divalent metal ions such as Fe2+, Mn2+, Cu2+, Zn2+, Cd2+, and Pb2+. It is possible that DMT1 also plays a role in the absorption of Cu. Therefore, maintaining an appropriate copper concentration is crucial for cellular function (34). The protein FKBP4, often referred to as FKBP52, is an immunophilin. Linked to HSP90, it aids in assembling various protein complexes (35, 36). Acting as a scaffold, FKBP4 promotes interactions among key components of multiple cancer-promoting signaling pathways (37, 38). FKBP52 is a constituent of the copper efflux mechanism, potentially contributing to neuroprotection against copper toxicity (39). AANAT serves as the rate-limiting enzyme in melatonin synthesis. However, it is unclear the role of AANAT in copper metabolism. According to current study, O-GlcNAcylation of YY1 targets SLC22A15 and AANAT, which promotes carcinogenesis in colorectal cancer cells (40). To sum up, our results showed that TFRC, SORD, SLC11A2, FKBP4, and AANAT maybe novel genes played significant roles in Ewing’s sarcoma.

A nomogram visually presents a prediction model, forecasting a patient’s prognosis based on clinical features. It also identifies significant prognosis-affecting factors by comparing patient survival rates. The risk score was validated as an independent prognostic factor by both univariate and multivariate Cox regression analysis. Subsequently, a nomogram was constructed for convenient ES prognosis prediction. The accuracy of survival prognosis prediction for ES was evaluated by calibration curves at 1, 3, and 5 years.

We use ssGSEA analysis to explore the functions of PR-CMRGs in different risk groups. Remarkably, a significant number of pathways connected to the immune system showed enrichment in the low-risk group. Copper chelation in tumor cells increased CD8+ T and NK cell influx, resulting in slowing tumor growth (41). Consequently, we hypothesized a close connection between copper metabolism and anti-tumor immunity. We then proceeded to scrutinize the variance in the TME between the two groups. Notably, augmented levels of immune cell infiltration correlated with a more favorable prognosis.

Given that the levels of immune checkpoints can predict immunotherapy response (42), we conducted a more in-depth examination of the differences in the levels of thirteen immunological checkpoints in the two groups. Our findings unveiled that NU.7441 and ABT.263 were benefit for the low-risk group, while AKT inhibitor VIII, AS601245, AUY922, Bleomycin, Tipifarnib, PHA.665752, MG.132, JNK.9L, BMS.708163, Erlotinib, and Imatinib showed greater advantages for the high-risk group. These results collectively pointed towards the potential of PR-CMRGs to offer guidance for immunotherapy in individuals diagnosed with ES.

Nonetheless, this study remains certain limitations merit. Initially, PR-CMRGs construction and validation depend on public databases, necessitating subsequent validation through future multicenter and prospective investigations. Secondly, further experimental endeavors are imperative to authenticate the individual and collective functions of the five genes encompassed within PR-CMRGs in ES.

To conclude, the study uncovered the significant influence of the interplay between copper metabolism and immunity on the progression of ES. For ES patients, the prognostic model based on 5 PR-CMRGs was developed and its prediction efficiency was well demonstrated. This model can be conducive to prognostic prediction and may provide a guidance on immunotherapy.

## Data availability statement

The original contributions presented in the study are included in the article/**Supplementary Material**. Further inquiries can be directed to the corresponding authors.

## Ethics statement

Ethical approval was not required for the studies on humans in accordance with the local legislation and institutional requirements because only commercially available established cell lines were used. Ethical approval was not required for the studies on animals in accordance with the local legislation and institutional requirements because only commercially available established cell lines were used.

## Author contributions

YC: Conceptualization, Formal analysis, Investigation, Writing – original draft, Writing – review & editing. WZ: Conceptualization, Formal analysis, Methodology, Writing – original draft, Writing – review & editing. XX: Data curation, Software, Validation, Writing – original draft, Writing – review & editing. BX: Data curation, Software, Validation, Writing – original draft, Writing – review & editing. YY: Data curation, Software, Validation, Writing – original draft, Writing – review & editing. HY: Data curation, Software, Validation, Visualization, Writing – original draft, Writing – review & editing. KL: Data curation, Software, Writing – original draft, Writing – review & editing. ML: Data curation, Software, Writing – original draft, Writing – review & editing. LQ: Conceptualization, Funding acquisition, Investigation, Methodology, Project administration, Supervision, Writing – original draft, Writing – review & editing. XJ: Conceptualization, Investigation, Methodology, Project administration, Supervision, Writing – original draft, Writing – review & editing.
