# Supplementary material for: Gene signatures of copper metabolism related genes may predict prognosis and immunity status in Ewing’s sarcoma
Source: Front Oncol. 2024 Jul 9;14:1388868. doi: 10.3389/fonc.2024.1388868 (PMC11267503; doi:10.3389/fonc.2024.1388868)
Supplement: Supplementary file 1 [file Table_1.docx]

Supplementary table 1. Copper metabolism-related genes

| Gene Symbol |  |  |  |  |  |
| --- | --- | --- | --- | --- | --- |
| ABCB6  ANKRD9  SLC31A1  SLC31A2  PRND  CCDC22  APP  ARF1  MT2A  ATOX1  ATP7A  ATP7B  PRNP  SCO1  COX19  SCO2  CYP1A1  DAXX  BACE1  AOC1  MT1DP  HSF1 | AQP1  AQP2  MT1A  MT1B  MT1E  MT1F  MT1G  MT1H  MT1M  MT1X  MT3  NFE2L2  MT1HL1  SNCA  MAP1LC3A  MT4  BECN1  COMMD1  XIAP  CUTC  STEAP2  STEAP3  STEAP4 | SLC11A2  COX17  CP  FKBP4  HEPHL1  MMGT1  HEPH  PARK7  AANAT  IL1A  LCAT  LOXL2  MT-CO1  PAM  ATP5F1D  SOD1  SOD3  SORD  TFRC  CDK1  MOXD2P  MTCO2P12  COX11 | LACC1  DBH  DCT  ALB  F5  F8  OR5AR1  ADNP  ATP13A2  MOXD1  GPC1  ANG  SUMF1  AOC2  SNAI3  APOA4  COA6  LOX  LOXL1  MT-CO2  ACR  P2RX4  CUTA | HAMP  S100A5  S100A12  S100A13  SNCB  SNCG  TP53  TYR  LOXL4  LOXL3  AOC3  RNF7  CCS  AP1S1  AP1B1  TMPRSS6  SPATA5  COG2  ATP6V0A2  ATP6AP1  ADAM10  AKT1  MTF2 | FOXO1  FOXO3  STEAP1  GSK3B  APC  JUN  MAPT  MDM2  MT1JP  MT1L  MTF1  PIK3CA  XAF1  PTEN  CCND1  SP1  ADAM17  CASP3  ADAM9 |
